# Supplementary material for: Benchmarking pipelines for subclonal deconvolution of bulk tumour sequencing data
Source: Nat Commun. 2021 Nov 4;12:6396. doi: 10.1038/s41467-021-26698-7 (PMC8569188; doi:10.1038/s41467-021-26698-7)
Supplement: Supplementary file 1 — Supplementary Information [file 41467_2021_26698_MOESM1_ESM.pdf]

SUPPLEMENTARY MATERIAL

Supplemental Tables

Supplemental Table 1. Precision and recall of the tested variant callers in target exon regions.
Supplemental Table 2. Number of true called variants and precision of the tested variant callers across the whole genome.
Supplemental Table 3. Precision and recall of the tested variant callers in target exon regions or across the whole genome for WES and WGS respectively for S2R2 at 100% purity and 100x.
Supplemental Table 4. Mean absolute difference of purity and ploidy estimates from true values, for each CNA caller and with different sequencing depths.
Supplemental Table 5. Mean absolute adjusted Rand index for pipeline CCF clustering shown in Figure 5.

Supplemental Figures

Supplemental Figure 1. Runtimes of variant calling pipelines when used on the S3R3_100%_250x sample.
Supplemental Figure 2. Heatmaps of true and predicted bulk copy numbers for total, minor, and major alleles.
Supplemental Figure 3. Accuracy of CCF estimates from pipelines run on samples with different variant and CNA frequencies, for a) non-clustered CCFs and b) clustered CCFs.
Supplemental Figure 4. Accuracy of CCF estimates from pipelines run on samples from the top performing tumours (S1R1 ,S2R1, S2R3) at 250x with varying tumour purities for a) non-clustered CCFs and b) clustered CCFs.
Supplemental Figure 5. Accuracy of CCF estimates from pipelines run on samples with varying tree topologies and numbers of clones, at 250x and 75% purity, for a) non-clustered CCFs and b) clustered CCFs.
Supplemental Figure 6. Accuracy of CCFs estimates from pipelines run on WGS of the best (S1R1) and worst (S2R2) performing WES tumours, at 100x and 75% purity for a) non-clustered CCFs and b) clustered CCFs.

Supplemental Table 1. Precision and recall of the tested variant callers in target exon regions.

Method	30x	60x	100x	250x
Precision in exon regions				
Strelka2	0.134	0.128	0.124	0.114
Strelka2_filtered	0.896	0.882	0.882	0.891
Lancet	0.135	0.074	0.049	0.028
Lancet_filtered	0.956	0.955	0.955	0.955
VarScan2	0.888	0.883	0.851	0.814
Varscan2_filtered	0.921	0.909	0.892	0.884
Mutect2	0.897	0.873	0.834	0.758
Mutect2_filtered	0.961	0.959	0.961	0.96
S2_fil-M2_fil_ic union	0.893	0.878	0.879	0.888
S2_fil-M2_fil_ic intersect	0.967	0.966	0.967	0.967
Recall in exon regions				
Strelka2	0.475	0.567	0.627	0.708
Strelka2_filtered	0.372	0.478	0.546	0.631
Lancet	0.367	0.492	0.579	0.689
Lancet_filtered	0.276	0.358	0.405	0.443
VarScan2	0.163	0.189	0.197	0.209
Varscan2_filtered	0.142	0.17	0.182	0.194
Mutect2	0.335	0.418	0.485	0.591
Mutect2_filtered	0.324	0.408	0.471	0.58
S2_fil-M2_fil_ic union	0.375	0.486	0.558	0.663
S2_fil-M2_fil_ic intersect	0.321	0.4	0.458	0.547
F1 score in exon regions				
Strelka2	0.209	0.209	0.208	0.197
Strelka2_filtered	0.526	0.62	0.674	0.738
Lancet	0.197	0.129	0.09	0.053
Lancet_filtered	0.428	0.521	0.569	0.605
VarScan2	0.276	0.312	0.321	0.332
Varscan2_filtered	0.246	0.287	0.303	0.319
Mutect2	0.488	0.566	0.614	0.664
Mutect2_filtered	0.484	0.572	0.632	0.723
S2_fil-M2_fil_ic union	0.528	0.626	0.683	0.759
S2_fil-M2_fil_ic intersect	0.482	0.565	0.622	0.699

Supplemental Table 2. Number of true called variants and precision of the tested variant callers across the whole genome.

Method	30x	60x	100x	250x
Number of true called variants across whole genome				
Strelka2	6356	8652	10517	14018
Strelka2_filtered	4771	6637	8011	10220
Lancet	4623	6811	8711	12138
Lancet_filtered	3267	4585	5511	6511
VarScan2	2067	2653	2970	3249
Varscan2_filtered	1763	2346	2686	3006
Mutect2	4316	5746	6798	8547
Mutect2_filtered	4161	5563	6546	8328
Precision across whole genome				
Strelka2	0.030	0.029	0.029	0.027
Strelka2_filtered	0.660	0.582	0.504	0.360
Lancet	0.118	0.063	0.040	0.020
Lancet_filtered	0.939	0.933	0.920	0.896
VarScan2	0.854	0.818	0.776	0.732
Varscan2_filtered	0.894	0.857	0.824	0.792
Mutect2	0.819	0.841	0.825	0.762
Mutect2_filtered	0.931	0.945	0.951	0.955

Supplemental Table 3. Precision and recall of the tested variant callers in target exon regions or across the whole genome for WES and WGS respectively for S2R2 at 100% purity and 100x.

Method	Precision		Recall	
	WES	WGS	WES	WGS
Strelka2	0.03	0.026	0.476	0.417
Strelka2_filtered	0.703	0.508	0.391	0.305
Lancet	0.004	0.006	0.451	0.452
Lancet_filtered	0.9	0.914	0.265	0.265
VarScan2	0.394	0.544	0.106	0.112
Varscan2_filtered	0.416	0.774	0.097	0.1
Mutect2	0.487	0.052	0.334	0.358
Mutect2_filtered	0.949	0.892	0.309	0.282

Supplemental Table 4. Mean absolute difference of purity and ploidy estimates from true values, for each CNA caller and with different sequencing depths for 75% and 100% purity samples.

Feature	Depth	Sequenza	FACETS	TITAN	Sclust
Purity	30x	0.004	0.060	0.138	0.019
	60x	0.041	0.047	0.143	0.016
	100x	0.041	0.043	0.088	0.02
	250x	0.009	0.044	0.064	0.014
	Combined	0.024	0.048	0.108	0.018
Ploidy	30x	0.548	0.590	0.628	0.729
	60x	0.818	0.592	0.579	0.729
	100x	1.034	0.588	0.610	0.730
	250x	0.69	0.591	0.606	0.612
	Combined	0.773	0.590	0.605	0.700

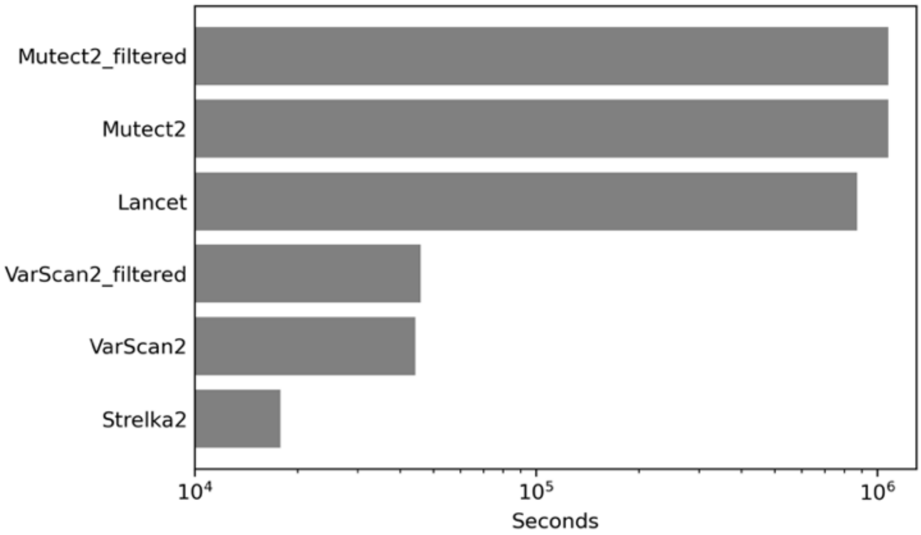

Supplemental Figure 1. Runtimes of variant calling pipelines when used on the S3R3_100%_250x sample. *Values include the time required to run additional programs necessary for each method (VarScan2 includes runtimes for GATK indel realignment and samtools mpileup, VarScan2_filtered further includes bam-readcount and fpfilter). Strelka2_filtered and Lancet_filtered do not require additional methods to acquire filtered variants and are therefore not indicated.*

Supplemental Figure 2. (Below) Heatmaps of true and predicted bulk copy numbers for total, minor, and major alleles. *FACETS, TITAN, and Sclust copy numbers are multiplied by the predicted cellular fractions containing them, and then added to diploid copy number values multiplied by the remaining cell fraction, thereby making them directly comparable to bulk values. Ground truth somatic copy number profiles were calculated by taking the ratios of somatic copy numbers to germline copy numbers.*

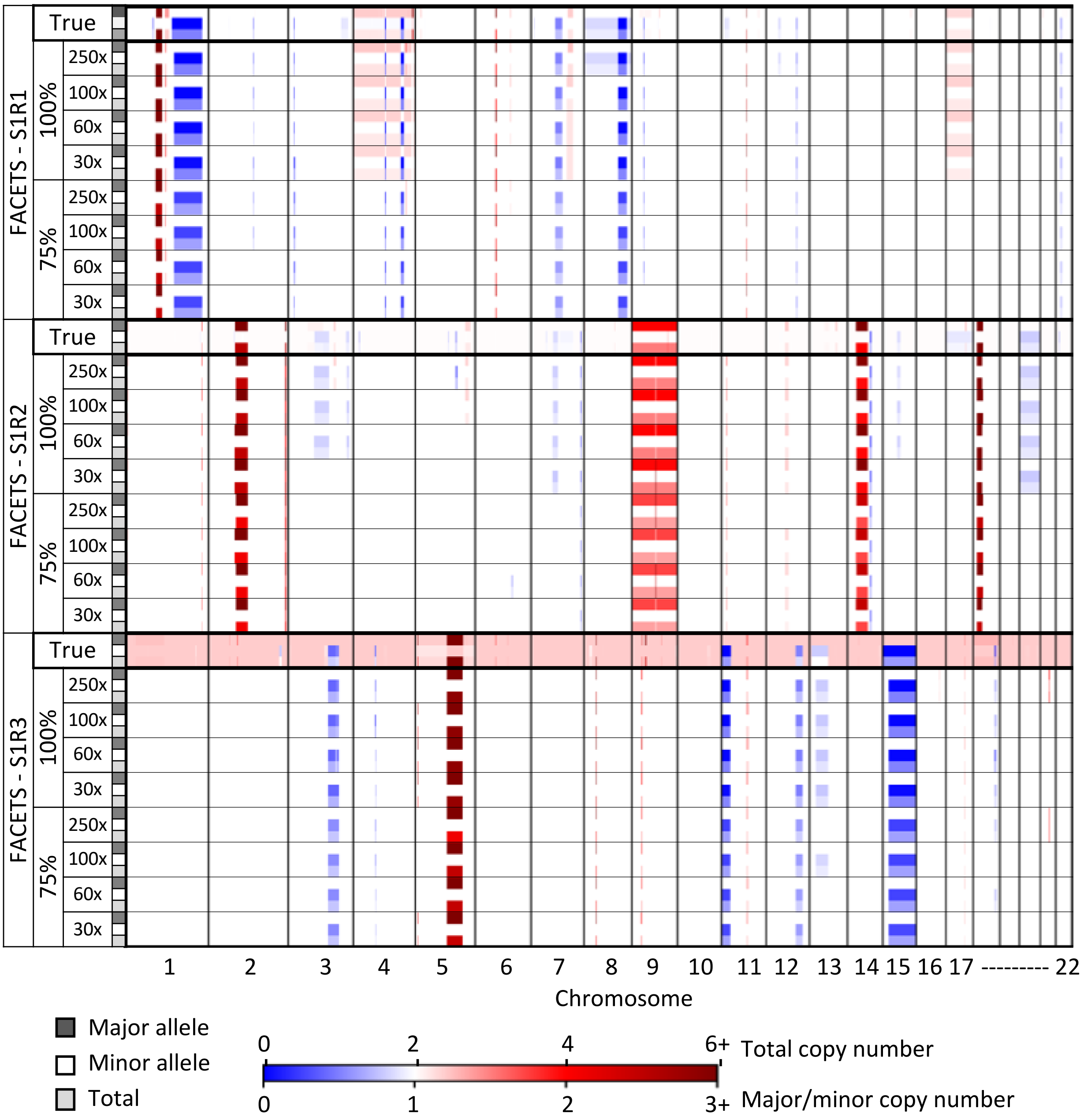

FACETS - S1R1
FACETS - S1R2
FACETS - S1R3
True
100%
75%
250x
100x
60x
30x
1 2 3 4 5 6 7 8 9 10 11 12 13 14 15 16 17 --------- 22
Chromosome
Major allele
Minor allele
Total
0 2 4 6+ Total copy number
0 1 2 3+ Major/minor copy number

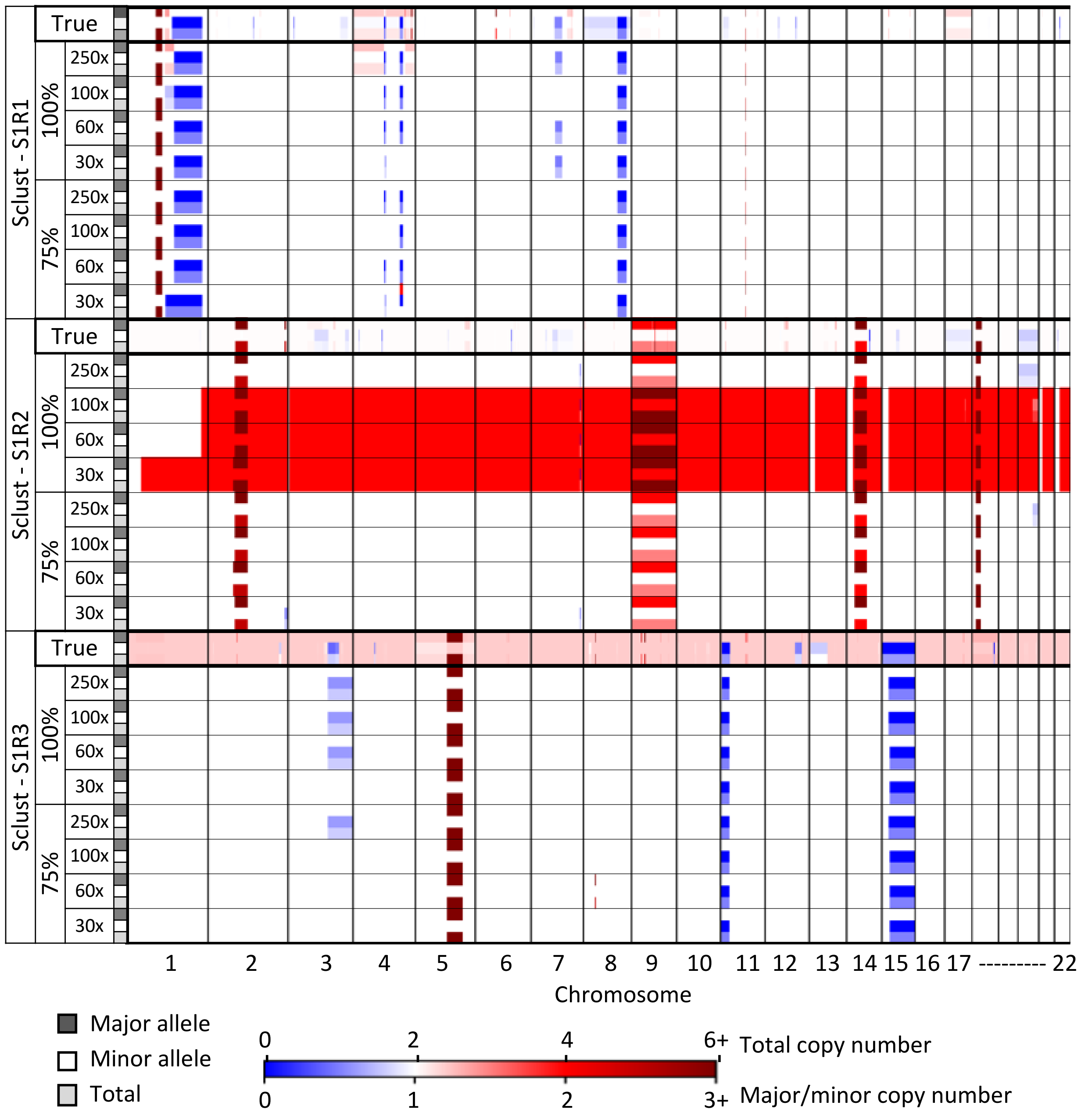
Sclust - S1R1
Sclust - S1R2
Sclust - S1R3
True
100%
75%
250x
100x
60x
30x
1
2
3
4
5
6
7
8
9
10
11
12
13
14
15
16
17
--------- 22
Chromosome
Major allele
Minor allele
Total
0
2
4
6+
Total copy number
0
1
2
3+
Major/minor copy number

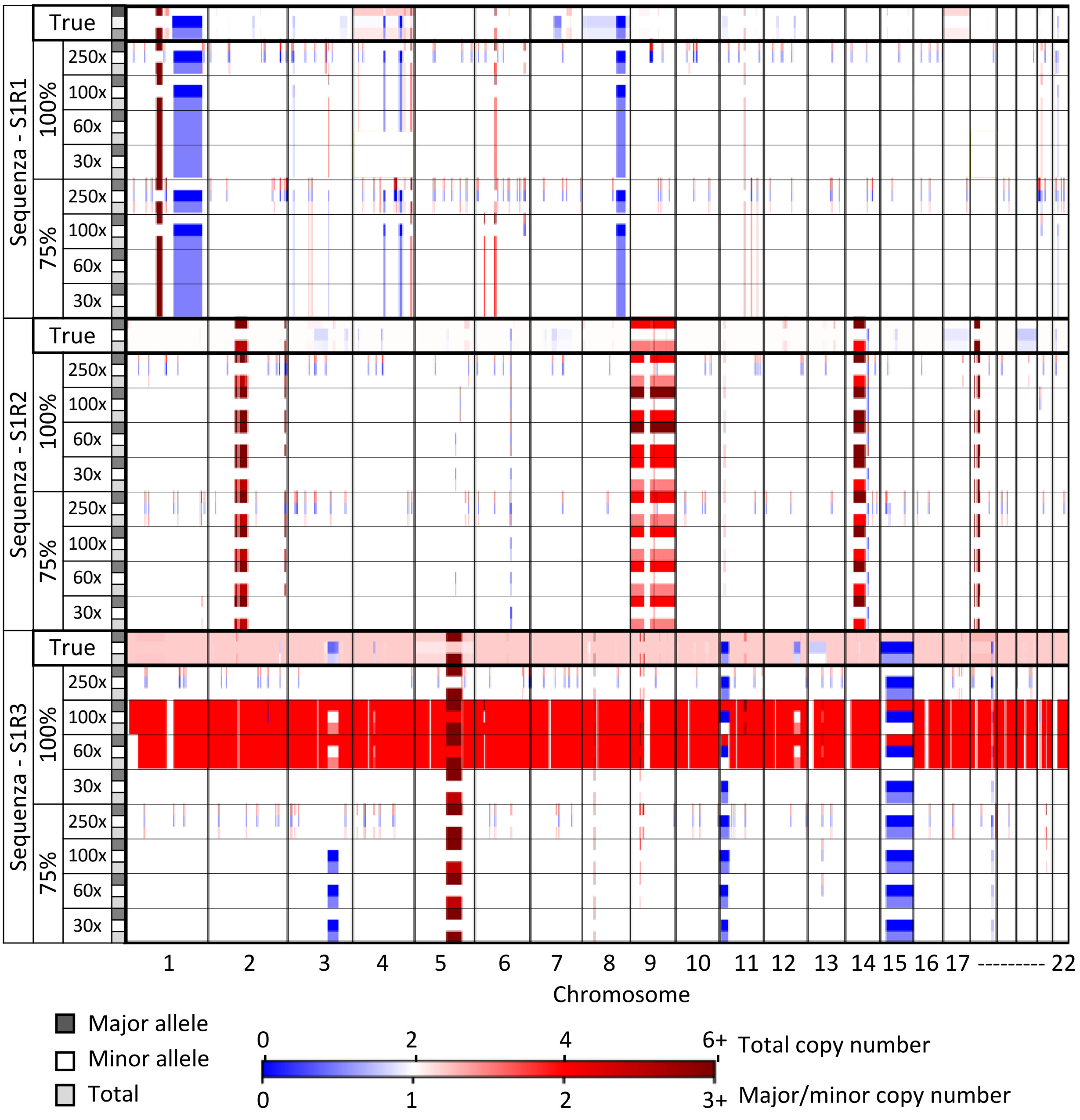

Sequenza - S1R1
Sequenza - S1R2
Sequenza - S1R3
True
100%
75%
250x
100x
60x
30x
1
2
3
4
5
6
7
8
9
10
11
12
13
14
15
16
17
--------- 22
Chromosome
Major allele
Minor allele
Total
0
2
4
6+
Total copy number
0
1
2
3+
Major/minor copy number

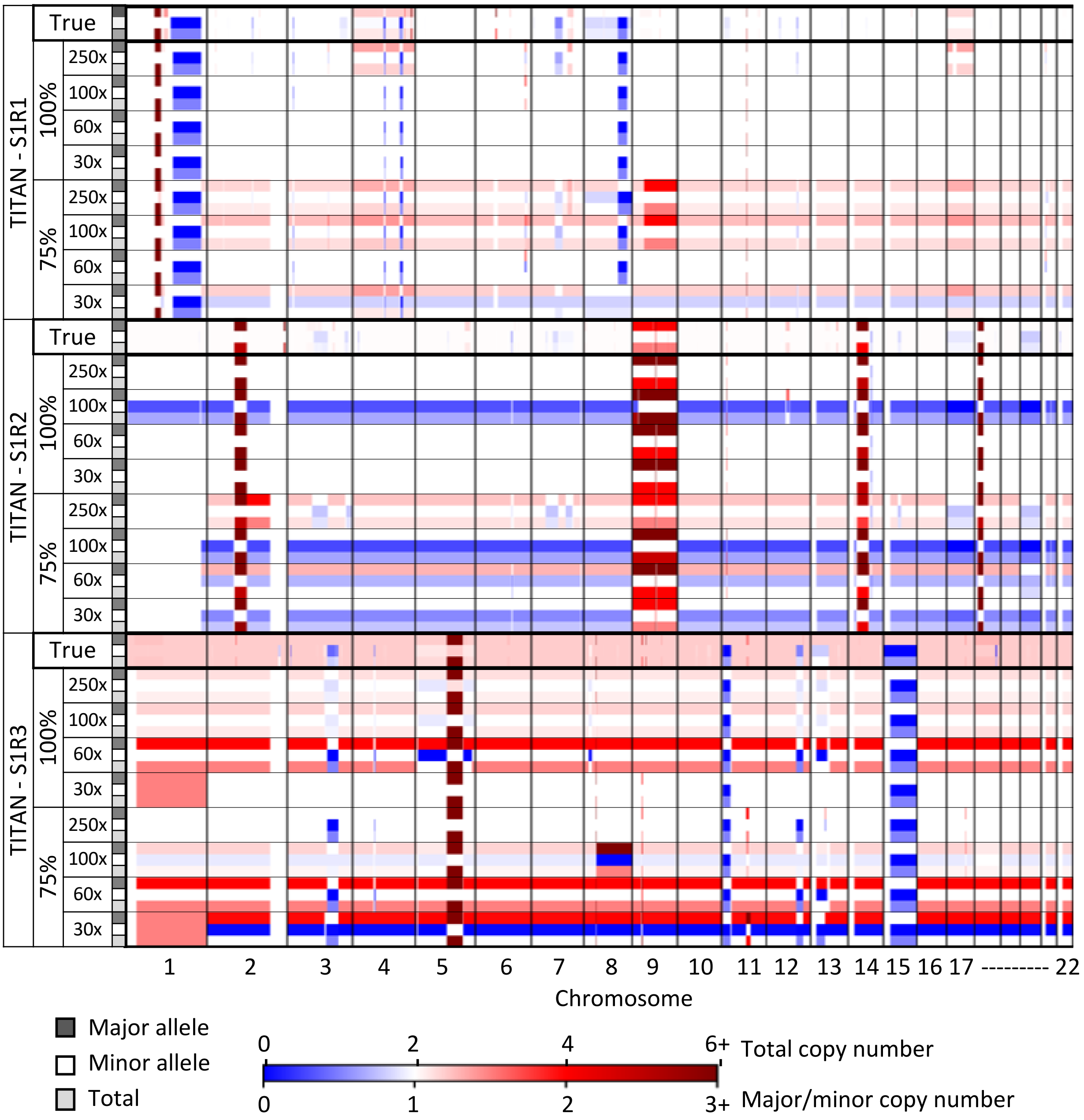
TITAN - S1R1
TITAN - S1R2
TITAN - S1R3
True
100%
75%
250x
100x
60x
30x
1 2 3 4 5 6 7 8 9 10 11 12 13 14 15 16 17 --------- 22
Chromosome
Major allele
Minor allele
Total
0 2 4 6+ Total copy number
0 1 2 3+ Major/minor copy number

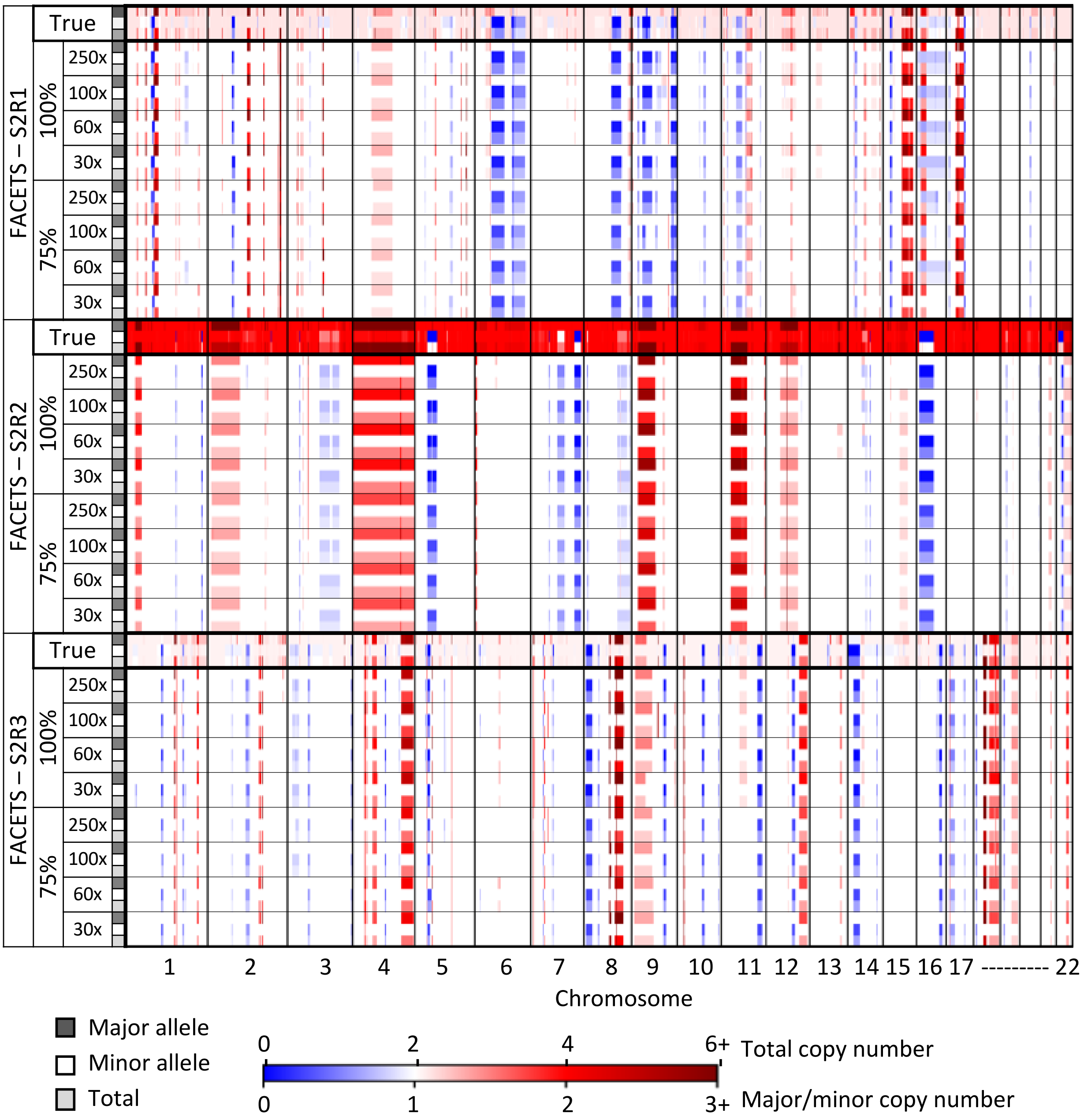

FACETS – S2R1
FACETS – S2R2
FACETS – S2R3
True
100%
75%
250x
100x
60x
30x
1
2
3
4
5
6
7
8
9
10
11
12
13
14
15
16
17
--------- 22
Chromosome
Major allele
Minor allele
Total
0
2
4
6+
Total copy number
0
1
2
3+
Major/minor copy number

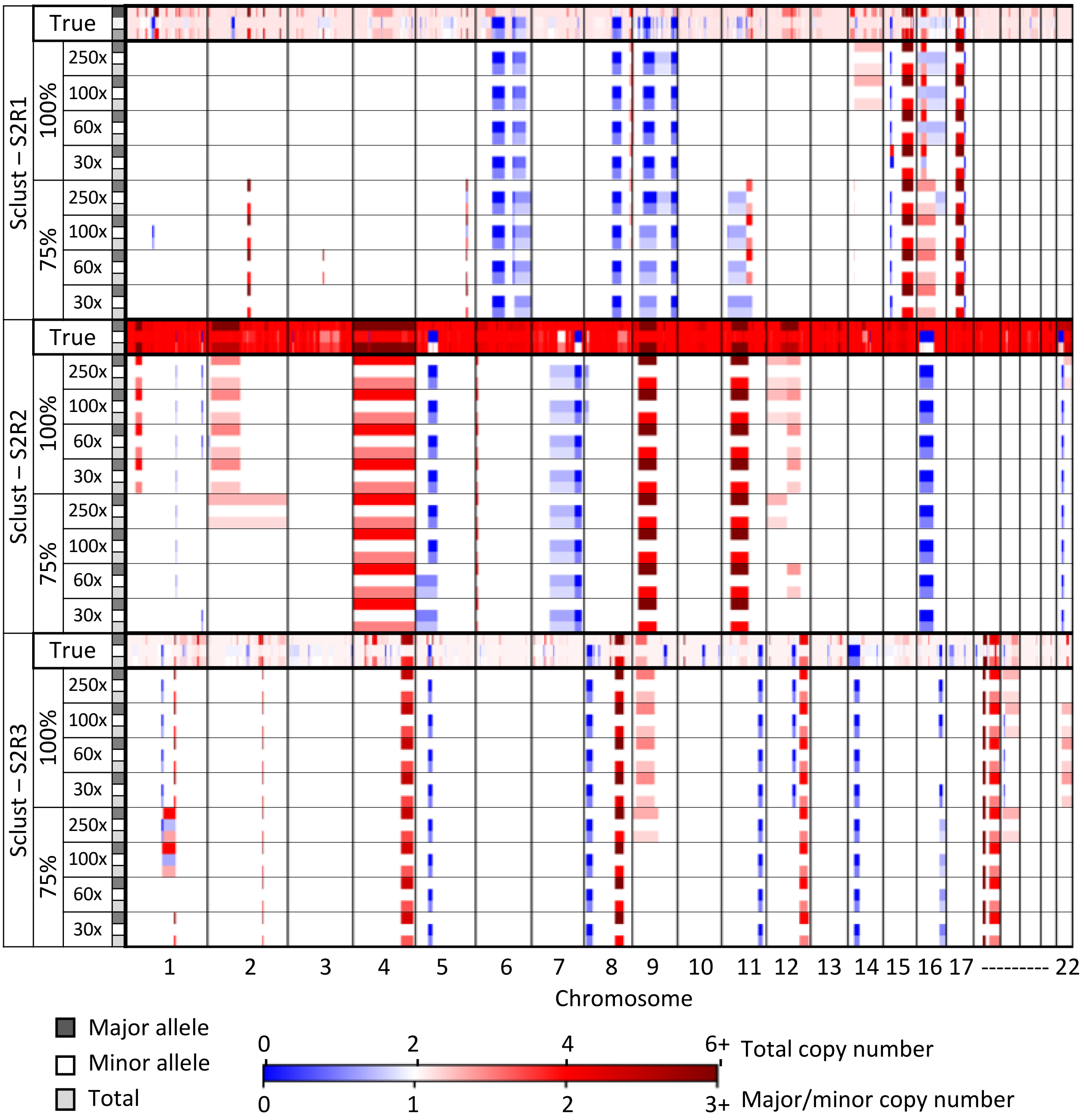

Sclust – S2R1
Sclust – S2R2
Sclust – S2R3
True
100%
75%
250x
100x
60x
30x
1
2
3
4
5
6
7
8
9
10
11
12
13
14
15
16
17
--------- 22
Chromosome
Major allele
Minor allele
Total
0
2
4
6+
Total copy number
0
1
2
3+
Major/minor copy number

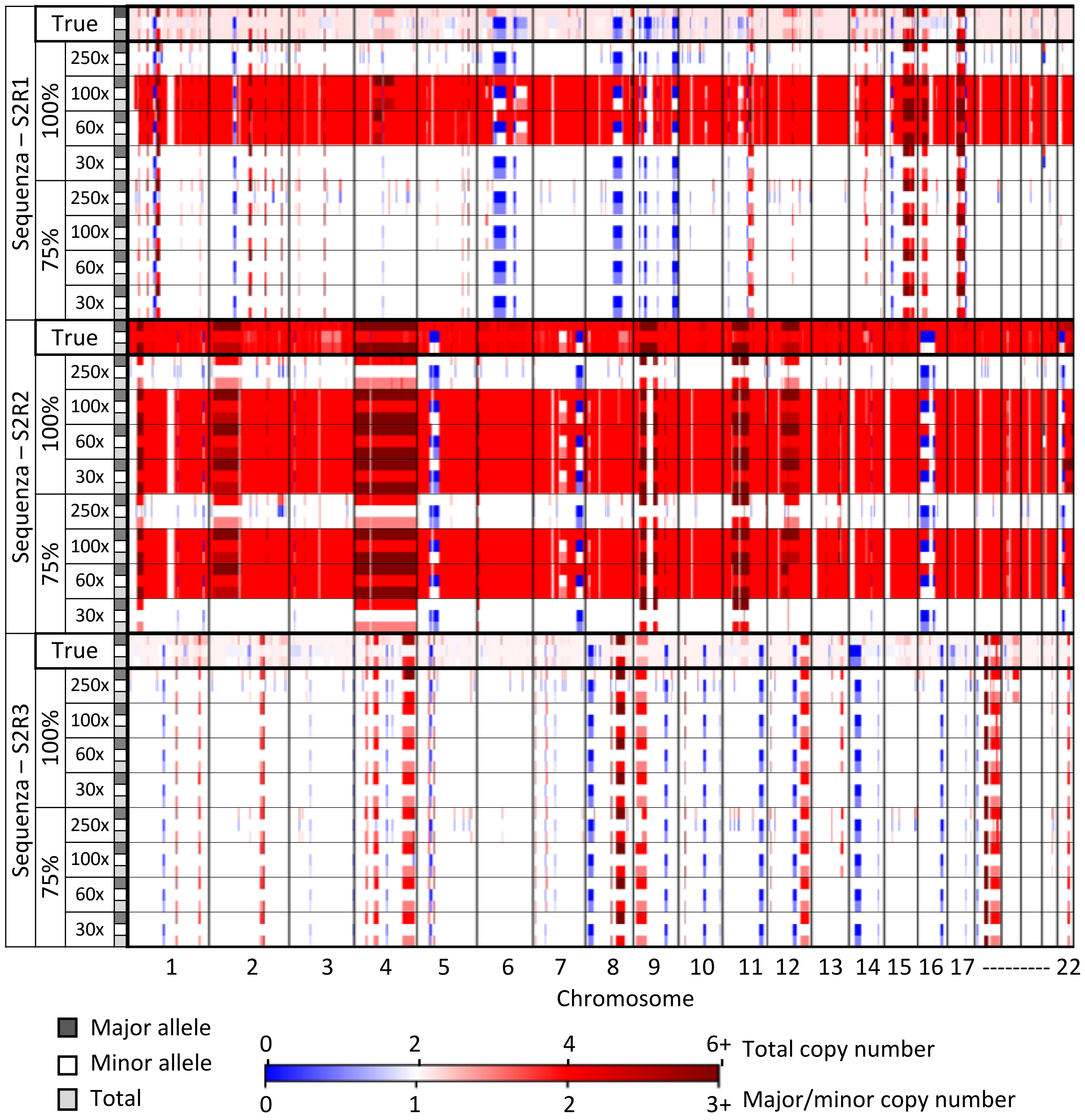

Sequenza – S2R1
Sequenza – S2R2
Sequenza – S2R3
True
100%
75%
250x
100x
60x
30x
1 2 3 4 5 6 7 8 9 10 11 12 13 14 15 16 17 --------- 22
Chromosome
Major allele
Minor allele
Total
0 2 4 6+ Total copy number
0 1 2 3+ Major/minor copy number

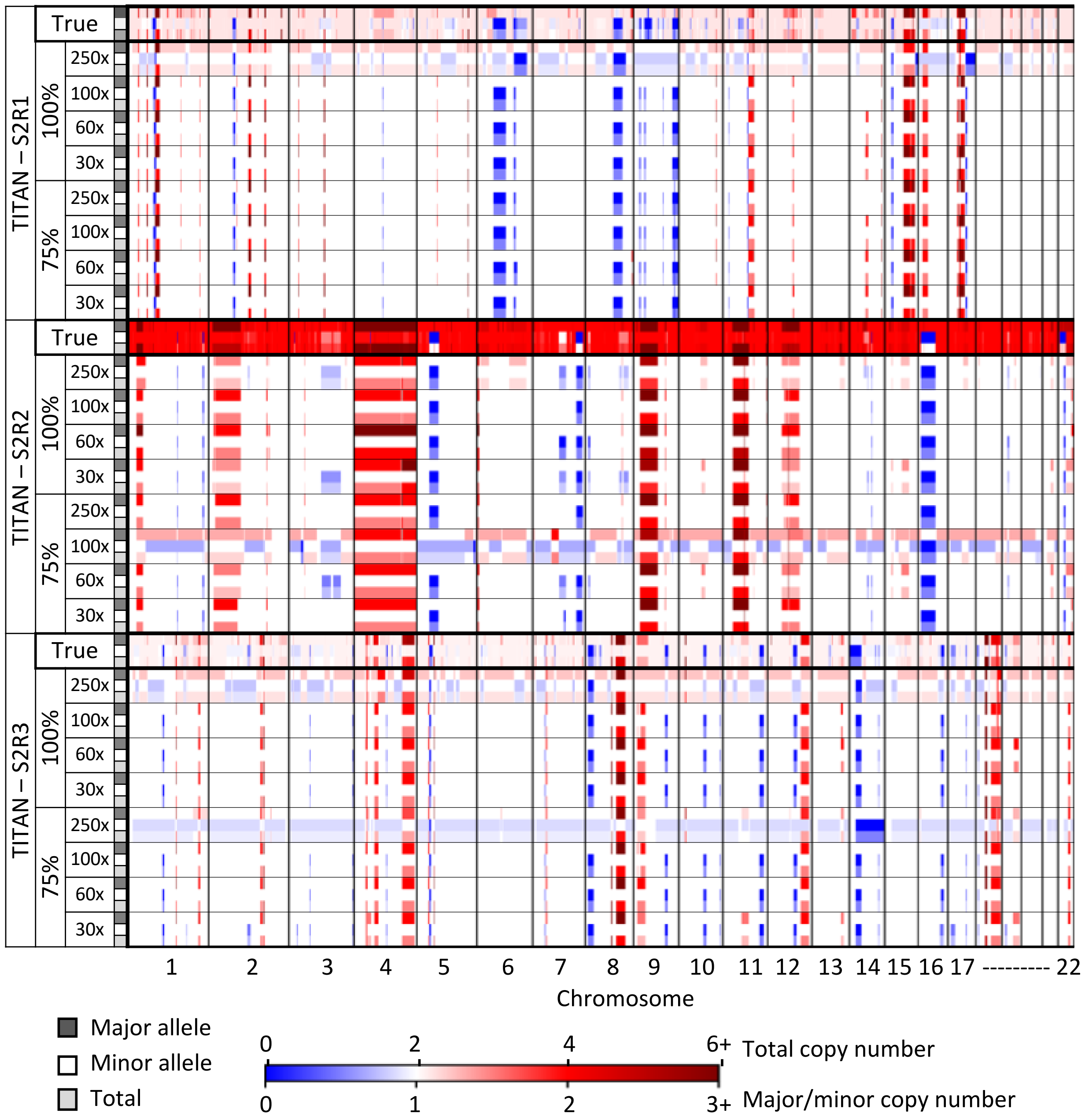

TITAN – S2R1
TITAN – S2R2
TITAN – S2R3
True
100%
75%
250x
100x
60x
30x
1 2 3 4 5 6 7 8 9 10 11 12 13 14 15 16 17 --------- 22
Chromosome
Major allele
Minor allele
Total
0 2 4 6+ Total copy number
0 1 2 3+ Major/minor copy number

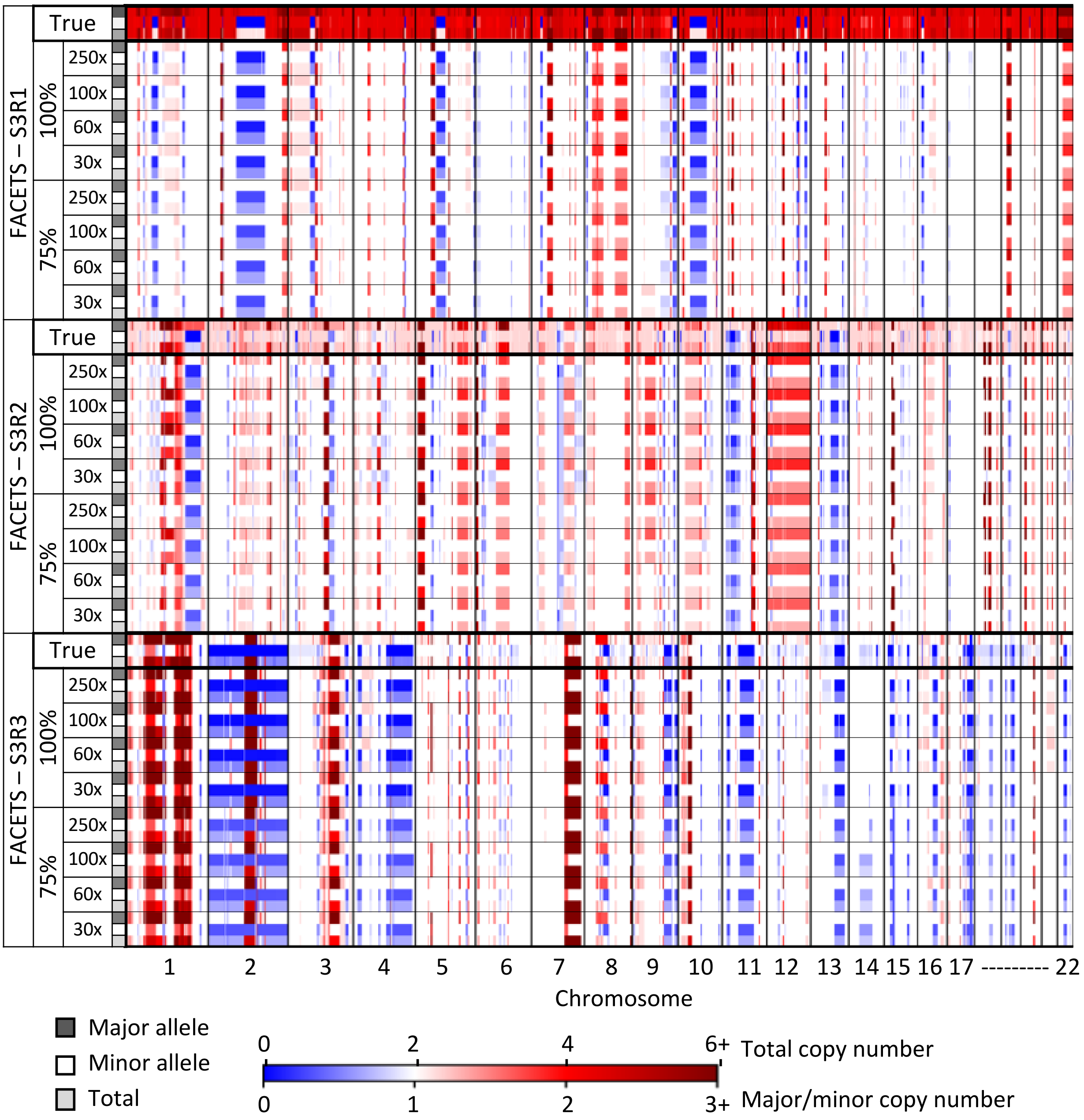

FACETS – S3R1
FACETS – S3R2
FACETS – S3R3
True
100%
75%
250x
100x
60x
30x
1
2
3
4
5
6
7
8
9
10
11
12
13
14
15
16
17
--------- 22
Chromosome
Major allele
Minor allele
Total
Total copy number
0
2
4
6+
Major/minor copy number
0
1
2
3+

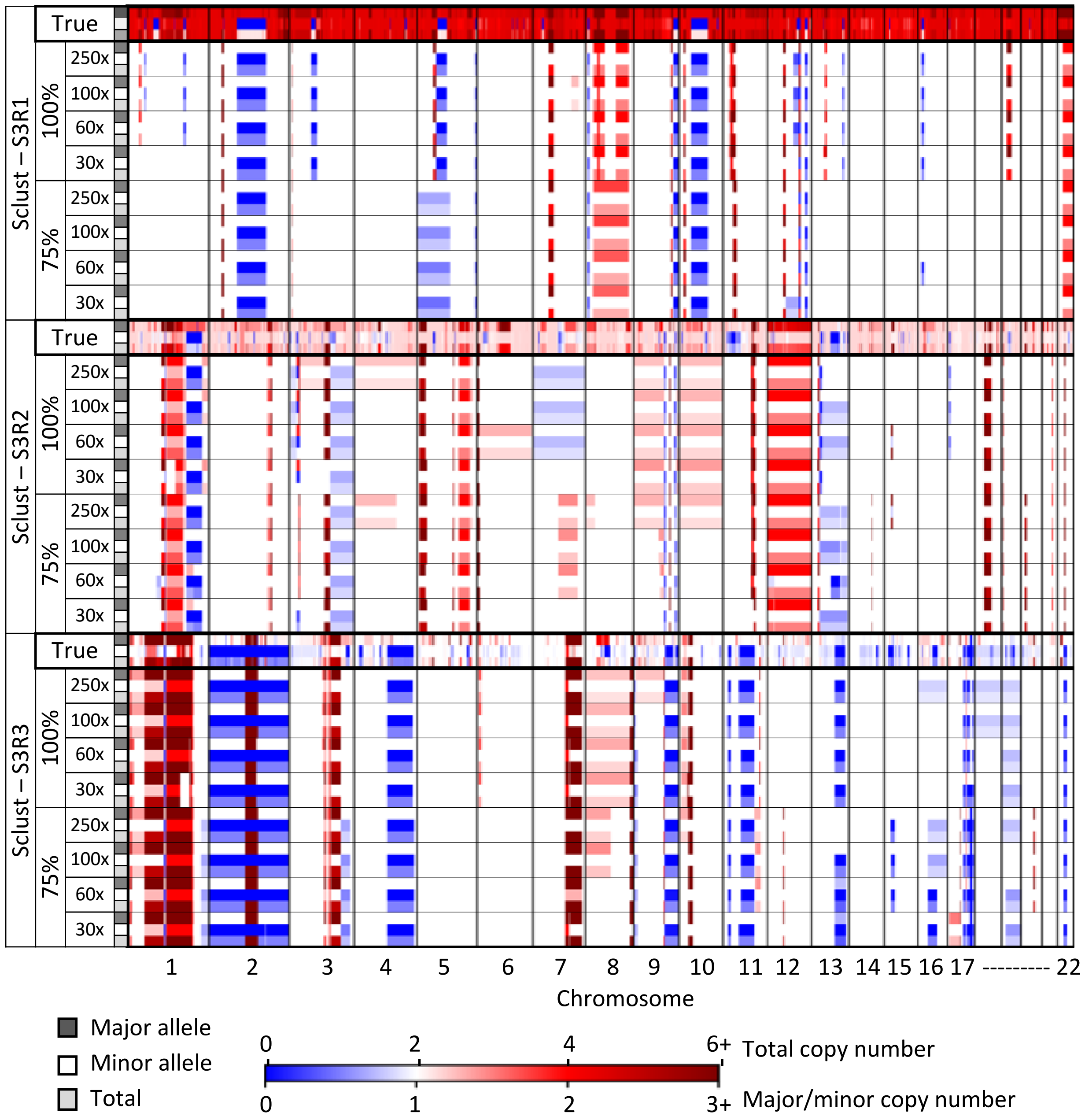

Sclust – S3R1
Sclust – S3R2
Sclust – S3R3
True
100%
75%
250x
100x
60x
30x
1
2
3
4
5
6
7
8
9
10
11
12
13
14
15
16
17
--------- 22
Chromosome
Major allele
Minor allele
Total
0
2
4
6+
Total copy number
0
1
2
3+
Major/minor copy number

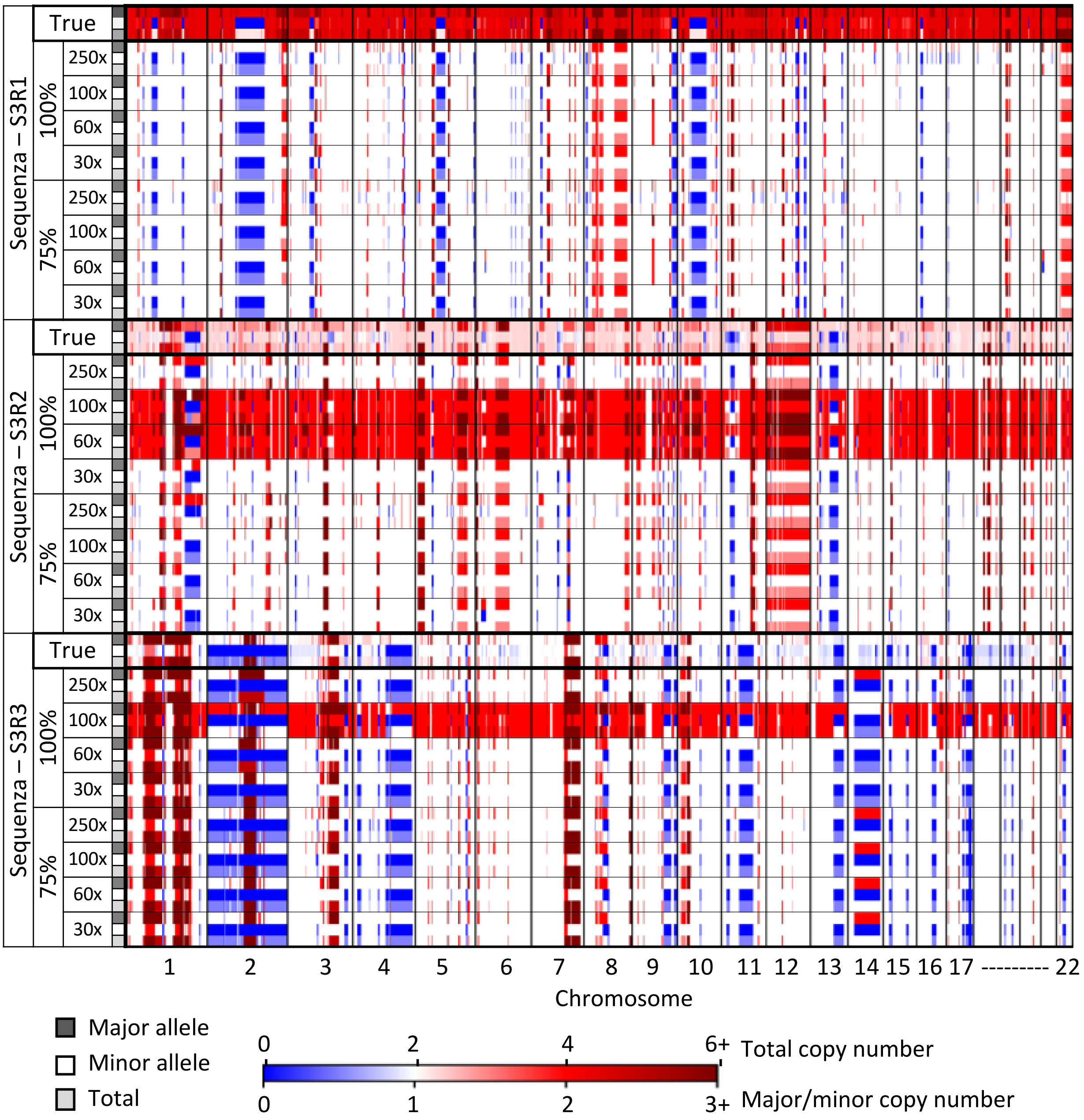
Sequenza – S3R1
Sequenza – S3R2
Sequenza – S3R3
True
100%
75%
250x
100x
60x
30x
1
2
3
4
5
6
7
8
9
10
11
12
13
14
15
16
17
--------- 22
Chromosome
Major allele
Minor allele
Total
0
2
4
6+
Total copy number
0
1
2
3+
Major/minor copy number

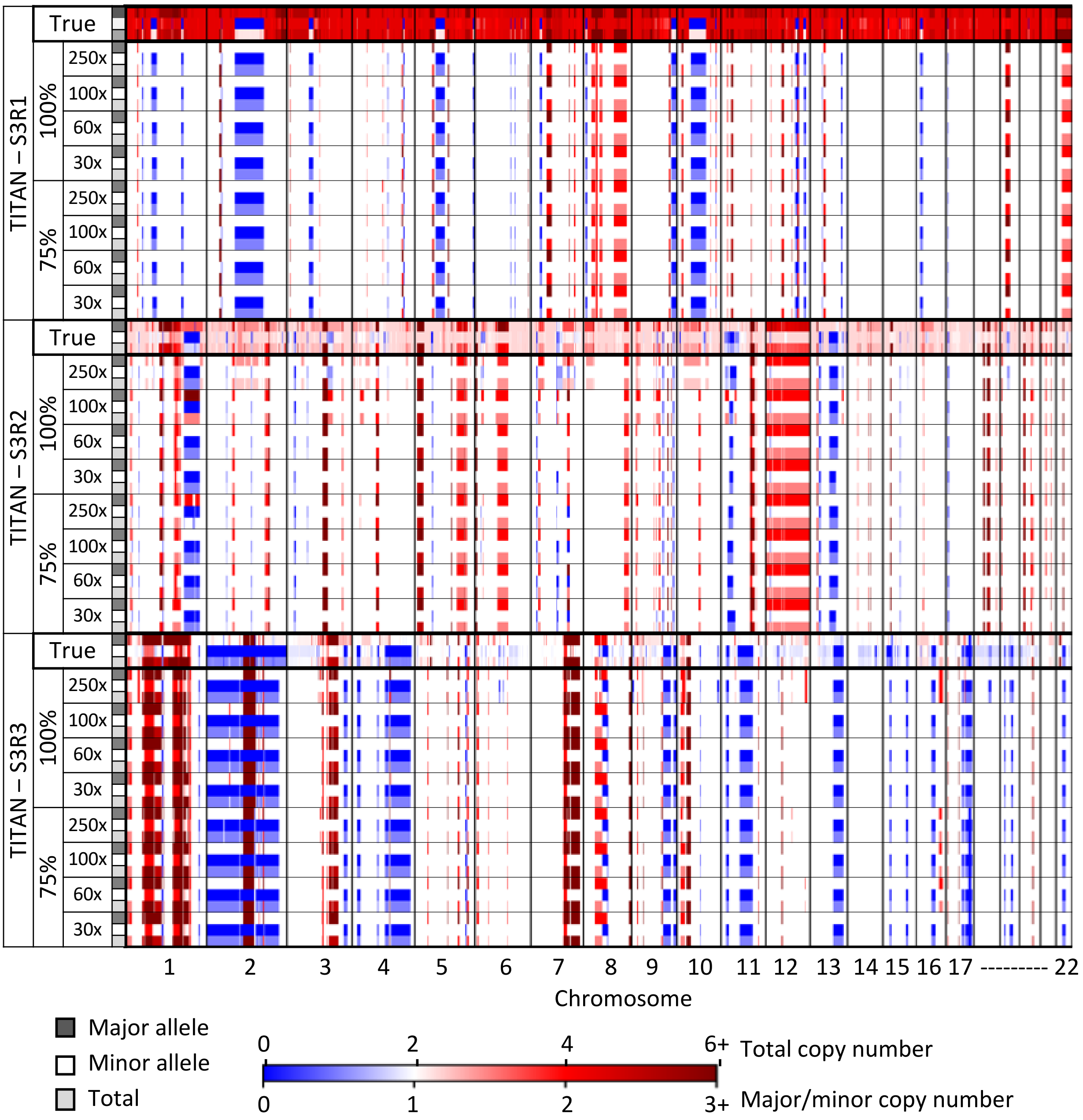
TITAN – S3R1
TITAN – S3R2
TITAN – S3R3
True
100%
75%
250x
100x
60x
30x
1 2 3 4 5 6 7 8 9 10 11 12 13 14 15 16 17 --------- 22
Chromosome
Major allele
Minor allele
Total
0 2 4 6+ Total copy number
0 1 2 3+ Major/minor copy number

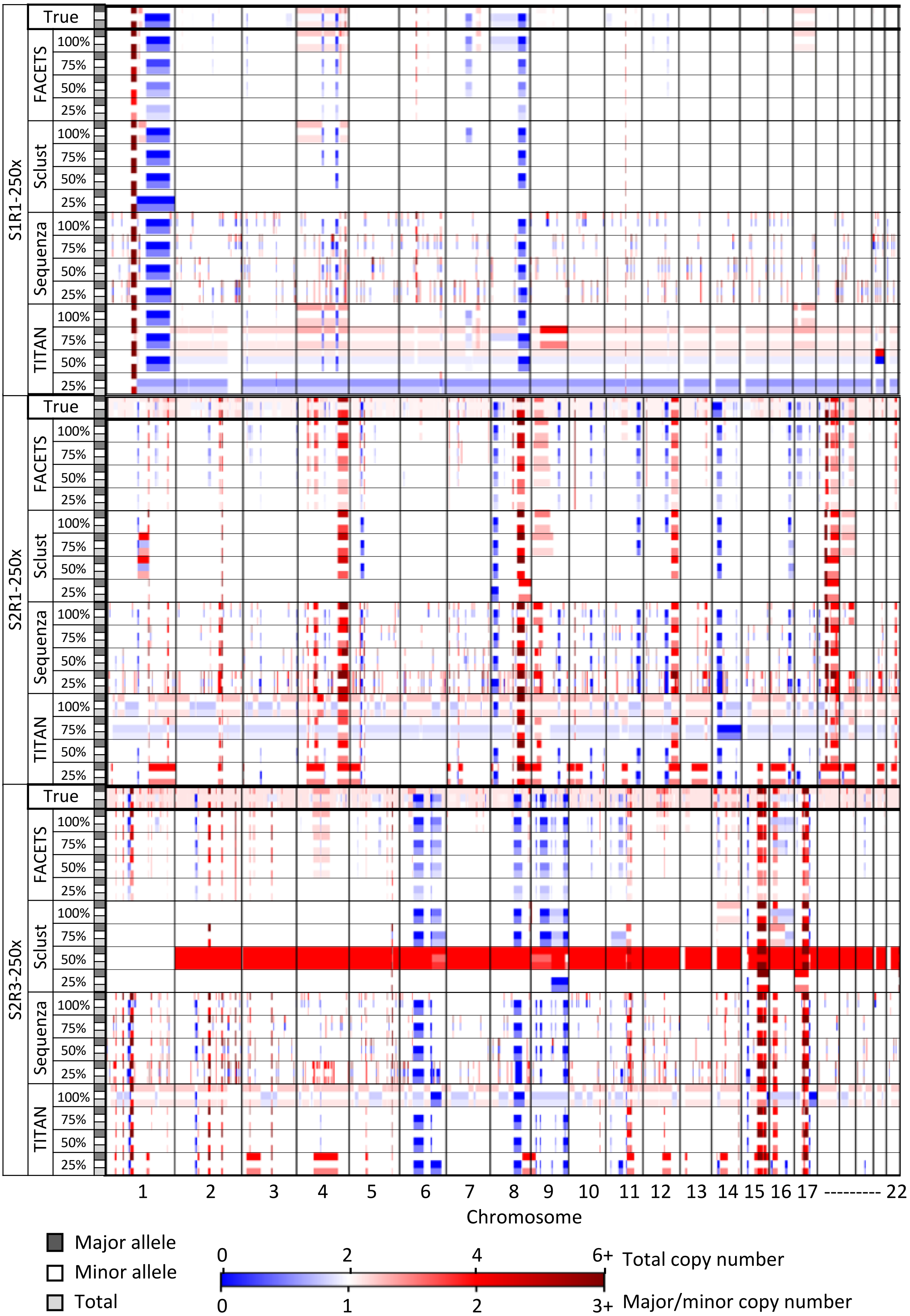

S1R1-250x
S2R1-250x
S2R3-250x
True
FACETS
Sclust
Sequenza
TITAN
100%
75%
50%
25%
1 2 3 4 5 6 7 8 9 10 11 12 13 14 15 16 17 --------- 22
Chromosome
Major allele
Minor allele
Total
0 2 4 6+ Total copy number
0 1 2 3+ Major/minor copy number

Supplemental Table 5. Mean absolute adjusted Rand index for pipeline CCF clustering shown in Figure 5.

Pipeline	30x	60x	100x	250x
FACETS Ccube	0.423	0.43	0.45	0.455
Sequenza Ccube	0.377	0.393	0.39	0.403
TITAN Ccube	0.336	0.368	0.375	0.413
FACETS FastClone	0.114	0.081	0.05	0.082
Sequenza FastClone	0.102	0.08	0.06	0.067
TITAN FastClone	0.121	0.099	0.065	0.081
FACETS PyClone	0.414	0.426	0.411	0.421
Sequenza PyClone	0.439	0.46	0.438	0.43
TITAN PyClone	0.326	0.362	0.366	0.338
FACETS PyClone-VI_binomial	0.431	0.453	0.447	0.465
Sequenza PyClone-VI_binomial	0.41	0.432	0.4	0.427
TITAN PyClone-Vi_binomial	0.365	0.396	0.403	0.425
FACETS PyClone-VI_beta-binomial	0.427	0.453	0.478	0.473
Sequenza PyClone-VI_beta-binomial	0.429	0.453	0.447	0.456
TITAN PyClone-Vi_beta-binomial	0.374	0.395	0.422	0.437
Sclust	0.323	0.379	0.377	0.401
K-means	0.292	0.337	0.377	0.385

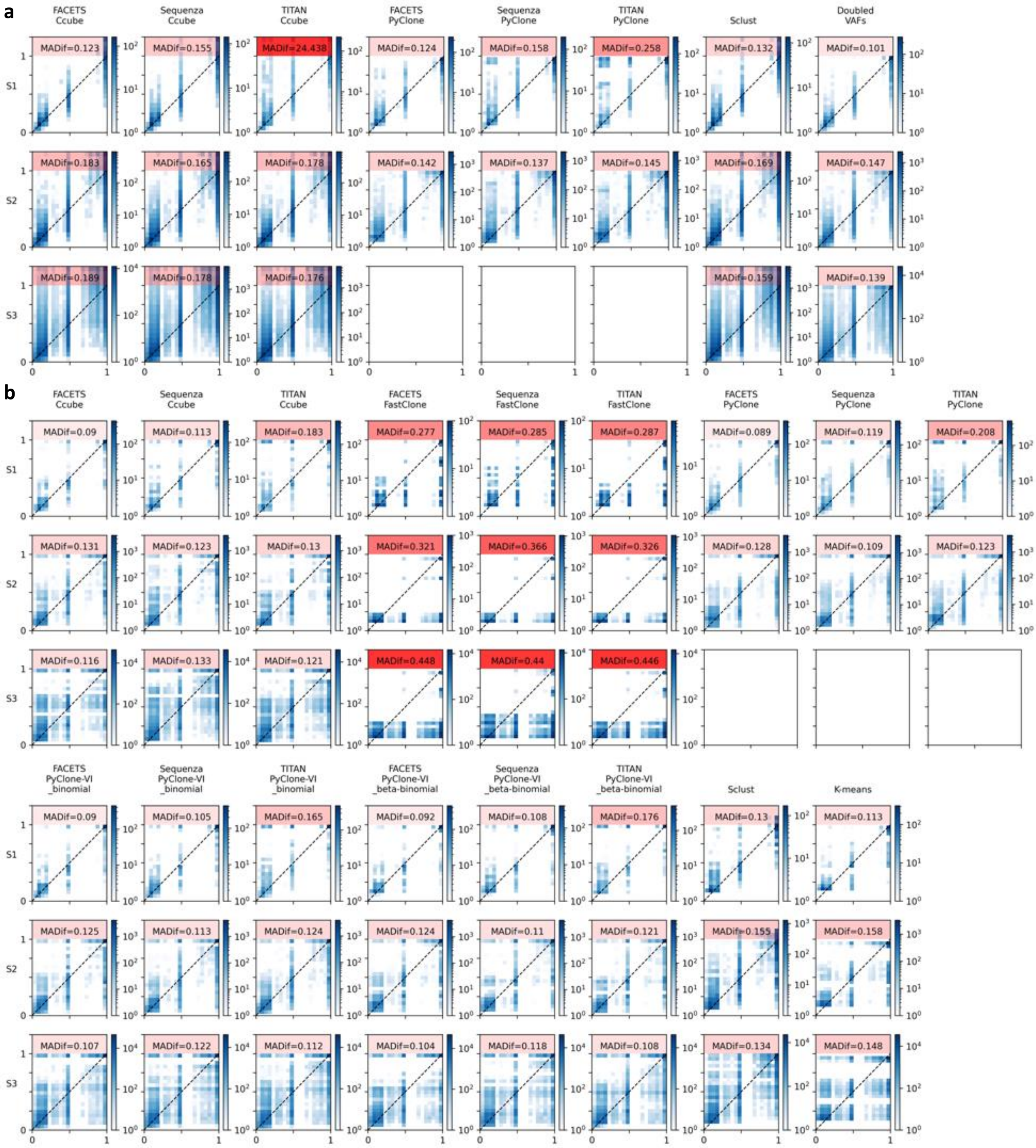

***Supplemental Figure 3*. Accuracy of CCF estimates from pipelines run on samples with different variant and CNA frequencies, for a) non-clustered CCFs and b) clustered CCFs.** *N=24 samples for each set. Estimates are limited to 1.25 in the heatmaps. Depths of red across MADifs illustrates their values. MADif: weighted mean absolute difference of true vs. estimated CCFs across variants in all samples in a group. S1: low variant and CNA frequency, S2: medium frequency, S3: high frequency. PyClone did not complete within two days for most S3 samples and is therefore not shown for these. FastClone did not fully converge for 61 of the 216 runs, and which are not included. The extreme MADif values for the TITAN_Ccube pipeline was due to an anomalous run where the mean estimated CCF was 607.03 as a result of TITAN predicting a purity of 0.0013% in a 100% pure sample. FastClone and PyClone-VI do not provide non-clustered CCF values.*

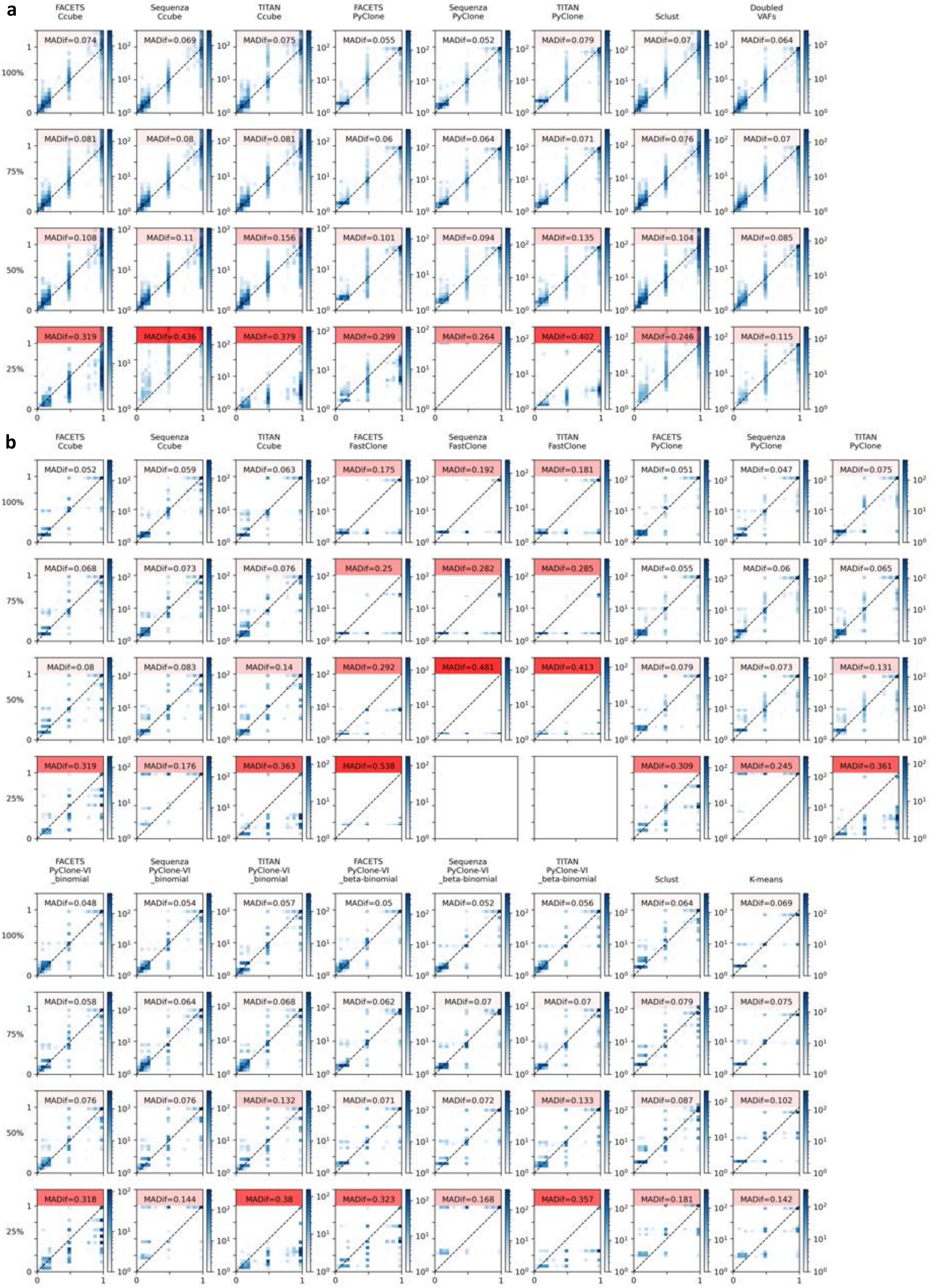

***Supplemental Figure 4**. **Accuracy of CCF estimates from pipelines run on samples from the top performing tumours (S1R1 ,S2R1, S2R3) at 250x with varying tumour purities for a) non-clustered CCFs and b) clustered CCFs.** N=3 samples for each purity. Estimates are limited to 1.25 in the heatmaps. Depths of red across MADifs illustrates their values. MADif: weighted mean absolute difference of true vs. estimated CCFs across variants in all samples in a group. FastClone did not fully converge for 11/36 runs, and which are not included. FastClone and PyClone-VI do not provide pre-clustered CCF values.*

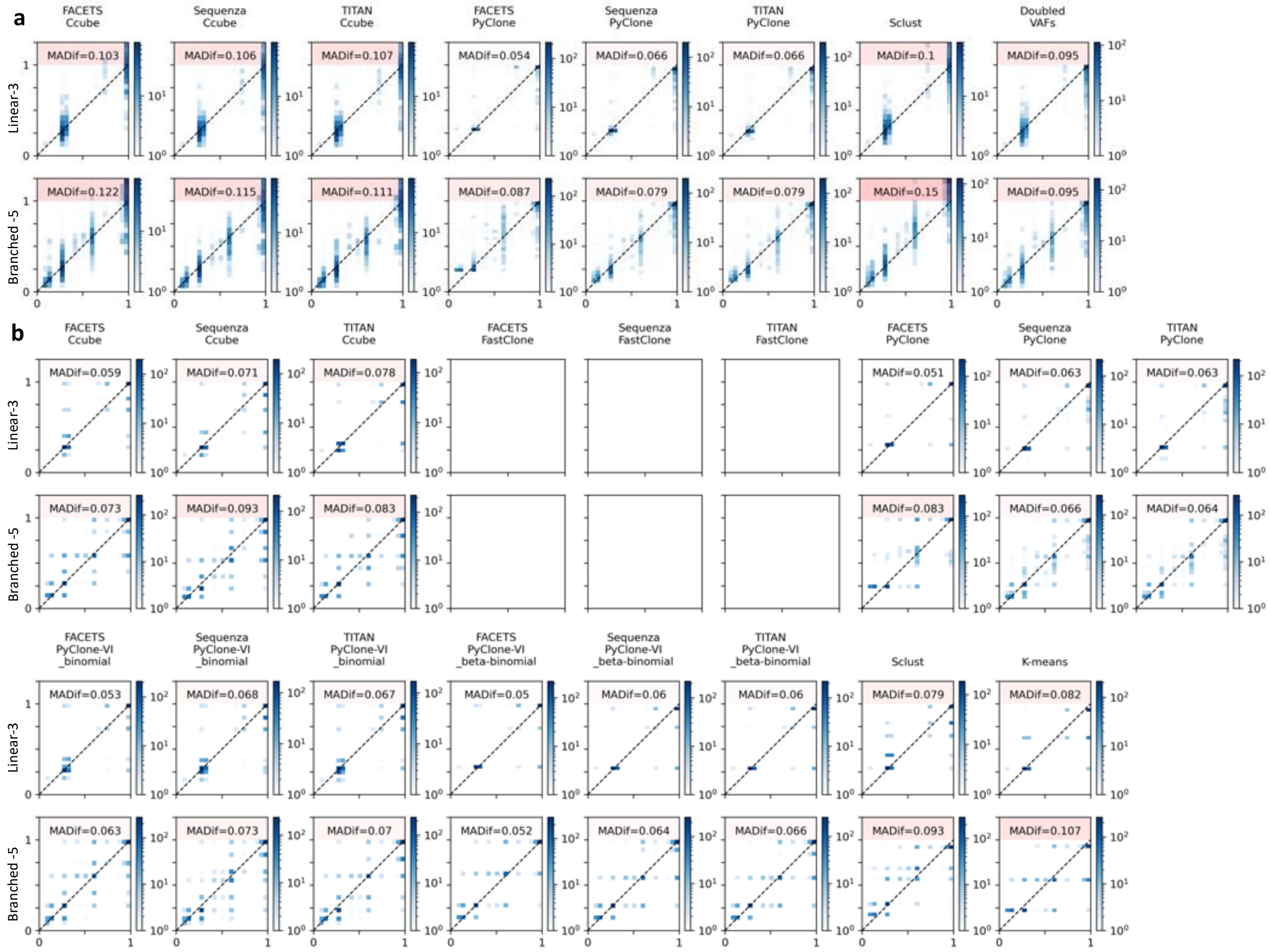

***Supplemental Figure 5**. Accuracy of CCF estimates from pipelines run on samples with varying tree topologies and numbers of clones, at 250x and 75% purity, for a) non-clustered CCFs and b) clustered CCFs.*
N=1 sample for each row. Estimates are limited to 1.25 in the heatmaps. Depths of red across MADifs illustrates their values. MADif: weighted mean absolute difference of true vs. estimated CCFs across variants in all samples in a group. FastClone did not fully converge on any runs and is therefore not shown. FastClone and PyClone-VI do not provide non-clustered CCF values.

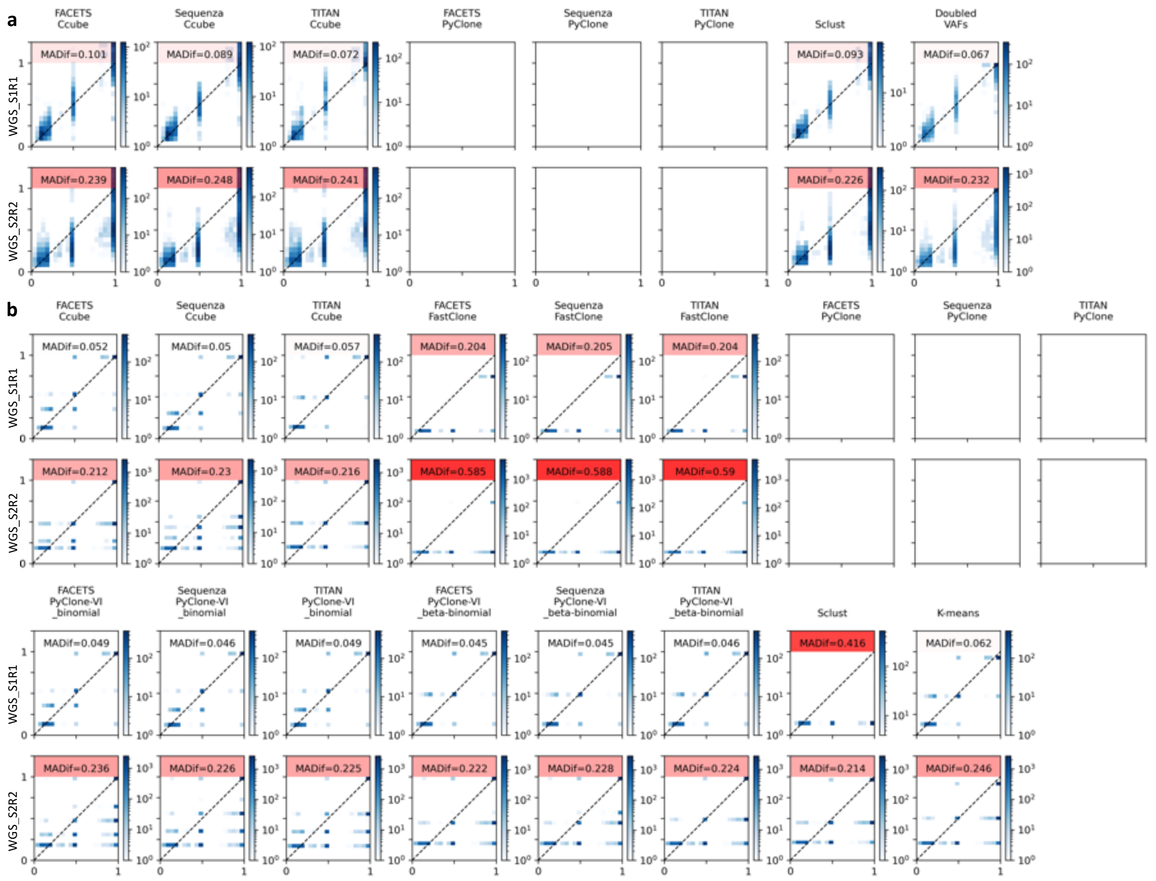

***Supplemental Figure 6**. Accuracy of CCFs estimates from pipelines run on WGS of the best (S1R1) and worst (S2R2) performing WES tumours, at 100x and 75% purity, for a) non-clustered CCFs and b) clustered CCFs.* *N=1 sample for each row. Estimates are limited to 1.25 in the heatmaps. Depths of red across MADifs illustrates their values. MADif: weighted mean absolute difference of true vs. estimated CCFs across variants in all samples in a group. PyClone did not complete within two days and is therefore not included. FastClone and PyClone-VI do not provide non-clustered CCF values.*
